# Risks and Benefits of Consumption of Great Lakes Fish

**DOI:** 10.1289/ehp.1003396

**Published:** 2011-09-23

**Authors:** Mary E. Turyk, Satyendra P. Bhavsar, William Bowerman, Eric Boysen, Milton Clark, Miriam Diamond, Donna Mergler, Peter Pantazopoulos, Susan Schantz, David O. Carpenter

**Affiliations:** 1 Division of Epidemiology and Biostatistics, School of Public Health, University of Illinois at Chicago, Chicago, Illinois, USA; 2 Environmental Monitoring and Reporting Branch, Ontario Ministry of the Environment, Toronto, Ontario, Canada; 3 Department of Environmental Science and Technology, University of Maryland, College Park, Maryland, USA; 4 Great Lakes Branch, Ontario Ministry of Natural Resources, Peterborough, Ontario, Canada; 5 Division of Environmental and Occupational Health Sciences, School of Public Health, University of Illinois at Chicago, Chicago, Illinois, USA; 6 Department of Geography and Program in Planning and Department of Chemical Engineering and Applied Chemistry, University of Toronto, Toronto, Ontario, Canada; 7 Centre de Recherche Interdisciplinaire sur la Biologie, la Santé, la Société et l’Environnement (CINBIOSE), Université du Québec à Montreal, Québec, Canada; 8 Food Laboratories Division, Health Canada, Ontario Region, Toronto, Ontario, Canada; 9 Department of Veterinary Biosciences, University of Illinois at Urbana-Champaign, Urbana, Illinois, USA; 10 Institute for Health and the Environment, University at Albany, Rensselaer, New York, USA

**Keywords:** dioxin, fish consumption, Great Lakes, methylmercury, omega-3 fatty acids, PCBs, risk assessment

## Abstract

Background: Beneficial effects of fish consumption on early cognitive development and cardiovascular health have been attributed to the omega-3 fatty acids in fish and fish oils, but toxic chemicals in fish may adversely affect these health outcomes. Risk–benefit assessments of fish consumption have frequently focused on methylmercury and omega-3 fatty acids, not persistent pollutants such as polychlorinated biphenyls, and none have evaluated Great Lakes fish consumption.

Objectives: The risks and benefits of fish consumption have been established primarily for marine fish. Here, we examine whether sufficient data are available to evaluate the risks and benefits of eating freshwater fish from the Great Lakes.

Methods: We used a scoping review to integrate information from multiple state, provincial, and federal agency sources regarding the contaminants and omega-3 fatty acids in Great Lakes fish and fish consumers, consumption rates and fish consumption advisories, and health effects of contaminants and omega-3 fatty acids.

Data synthesis: Great Lakes fish contain persistent contaminants—many of which have documented adverse health effects —that accumulate in humans consuming them. In contrast, data are sparse on omega-3 fatty acids in the fish and their consumers. Moreover, few studies have documented the social and cultural benefits of Great Lakes fish consumption, particularly for subsistence fishers and native communities. At this time, federal and state/provincial governments provide fish consumption advisories based solely on risk.

Conclusions: Our knowledge of Great Lakes fish has critical gaps, particularly regarding the benefits of consumption. A risk–benefit analysis requires more information than is currently available on the concentration of omega-3 fatty acids in Great Lakes fish and their absorption by fish eaters in addition to more information on the social, cultural, and health consequences of changes in the amount of fish consumed.

The relationship between the risks and benefits of fish consumption is complex. Consumption of fish and fish oils may be beneficial to early cognitive development and improve cardiovascular health in adults, but fish can also be an exposure route for toxic chemicals such as polychlorinated biphenyls (PCBs) and methylmercury, which confer risks for these same health outcomes (Castoldi et al. 2008; Kris-Etherton et al. 2003) and others (Buck Louis et al. 2009; Persky et al. 2001; Schell and Gallo 2010; Turyk et al. 2009).

Recommendations and advisories on fish consumption for both mercury and persistent organic pollutants (POPs) are often conflicting and can be confusing to consumers. The U.S. Food and Drug Administration (FDA) issued consumption advice for mercury in fish, but only for young children and women who are or might become pregnant or are nursing (U.S. FDA 2004). Health Canada advises different rates of consumption for the general population (150 g/week), for women who are or might become pregnant (150 g/month), for children 5–11 years of age (125 g/month), and for children 1–4 years of age (75 g/month) (Health Canada 2002). To add to the confusion, the American Heart Association advises consumption of at least two fish meals (7 ounces cooked) per week (American Heart Association 2010). They recommend fatty fish in particular, such as salmon, mackerel, herring, lake trout, sardines, and albacore tuna, but acknowledge that some types of fish may contain high levels of mercury, PCBs, dioxins, and other environmental contaminants.

Fish consumption advice has received considerable publicity in the popular press, and this has led to changes in consumption patterns. After the dissemination of advice by the U.S. FDA in 2001 recommending that women of childbearing age should avoid consuming specific long-lived predatory fish high in mercury and limit fish and shellfish meals, pregnant women in eastern Massachusetts decreased their total fish consumption, resulting in an estimated decline of 17% to 1.4 servings per month (Oken et al. 2003). A national sample of households suggested that fish consumption by those not targeted by the advisory also decreased, although it is not known if the participants were aware of the advisory per se (Shimshack et al. 2007). The health implications of changing a dietary protein source from fish to another source that may have less nutritional benefit are unclear. Fish, meat, poultry, and eggs are important sources of dietary protein, but fish generally have higher levels of selenium and omega-3 fatty acids and lower levels of saturated fats compared with other foods of animal origin (Institute of Medicine 2007).

The five Great Lakes have abundant fish stocks harvested by native peoples, recreational anglers, and commercial fishing enterprises. More than 160 aboriginal communities situated around the Great Lakes Basin have historically depended on local fish for a substantial proportion of their diet. Recreational Great Lakes sport fishing is a $7 billion annual industry in the United States (Southwick Associates 2008). In addition, lake whitefish, lake herring, yellow perch, walleye, chubs, smelt, lake trout, channel catfish, and carp all have commercial harvests. In 2000, the commercial harvest of lake whitefish was > 21 million pounds, and both the walleye and smelt harvests were > 7 million pounds, with a total harvest value of > $44 million (Kinnunen 2003). However, toxic substances related to agriculture, urbanization, industry, and transportation have caused a decline in Great Lakes water quality and contamination of Great Lakes fish with persistent, bioaccumulative, and toxic chemicals. This has triggered recommendations for restrictions on human fish consumption since the 1970s (Ashizawa et al. 2005; Tilden et al. 1997).

Currently, fish consumption advisories exist for some fish in all of the Great Lakes [Great Lakes Information Network 2010; Ontario Ministry of the Environment (OMOE) 2011]. In the United States, the Great Lakes states have developed a uniform fish consumption advisory approach that provides information on the amounts of fish that should be consumed over a particular period based on contaminant level, size, species, and location (Anderson et al. 1993, 2007). State fish advisories also include a statement recognizing that fish consumption has potential health benefits in comparison with other food sources. Advisories for the Canadian Great Lakes vary by lake and are related primarily to PCB levels and secondarily to dioxins/furans and mercury levels (Bhavsar et al. 2011). Ontario has also developed a comprehensive fish consumption advisory (OMOE 2011), which addresses both the health risks and benefits of fish consumption. Acknowledging that it is not possible to determine quantitatively the risk versus benefit of fish consumption, it concludes, “consumption of fish with high nutrient and low contaminant levels should be favoured.” The U.S. Environmental Protection Agency (EPA) has published general guidance for fish consumption based on contaminant concentrations (U.S. EPA 2000). Adherence to these guidelines would result in very limited or no consumption of most Great Lakes fish.

While a fair amount of information on contaminant concentrations in Great Lakes fish is available, little is known about the omega-3 fatty-acid content of these fish, creating significant uncertainty about how to balance the risks and benefits of eating Great Lakes fish. Indeed, most of our knowledge on the nutritional benefits of fish consumption is based on marine fish, which generally have higher concentrations of omega-3 fatty acids than freshwater fish (Li et al. 2011). Several reviews have focused on the benefits of nutrients and risks from contaminants in fish (e.g., Bushkin-Bedient and Carpenter 2010; Mozaffarian and Rimm 2006), but none have specifically examined this question with regard to Great Lakes fish. The objective of this publication is to review the data on contaminants and omega-3 fatty acids in Great Lakes fish, the concentrations of both fatty acids and contaminants found in persons who consume Great Lakes fish, and health effects of these factors. This is undertaken with the goal of identifying additional information that would be needed to undertake a risk–benefit analysis of Great Lakes fish consumption.

## Methods

We used a scoping approach (Anderson et al. 2008b) to contextualize knowledge with regard to the risks and benefits of Great Lakes fish consumption. The goal was not to undertake a systematic review of the literature relating to this topic, but rather to summarize a range of evidence from various sources in order to convey the breadth and depth of this issue. In a scoping review, a wide range of research and nonresearch material is synthesized to provide greater conceptual clarity about a topic or field of evidence. We identified relevant peer-reviewed literature, as well as government reports from state, federal, and provincial health departments, the U.S. EPA, the OMOE, and the International Joint Commission, containing relevant information on contaminants and omega-3 fatty acids in Great Lakes fish and fish consumers, information about fish consumption rates and fish advisories for Great Lakes fish, or information about health effects of contaminants or omega-3 fatty acids present in Great Lakes fish. We have included data on fish from the five Great Lakes, mouths of rivers feeding into the Great Lakes, and the St. Lawrence River.

## Contaminants and Great Lakes Fish

*Historical and current contaminants in Great Lakes fish.* The Great Lakes are contaminated with many bioaccumulative pollutants including PCBs, polychlorinated dibenzo dioxins and furans (PCDD/Fs), polybrominated diphenyl ethers (PBDEs), methylmercury, and chlorinated pesticides such as dichlorodiphenyltrichloroethane (DDT) and its metabolites. Most of these contaminants are lipophilic and therefore are found in higher concentrations in fatty, older, and piscivorous fish. Levels of methylmercury are also higher in older and predatory fish, but because methylmercury binds to protein, it bioaccumulates in muscle tissue rather than fatty tissue.

Contaminant levels measured in edible portions of a variety of Great Lakes fish are monitored by the OMOE (in collaboration with the Ontario Ministry of Natural Resources), by the U.S. states that border the Great Lakes, and by some tribal nations and are used to issue fish consumption advisories. In addition, the U.S. EPA and Environment Canada have ongoing, long-term efforts to monitor whole-body contaminant levels of top predator fish species (i.e., walleye in Lake Erie, lake trout in all other lakes) in order to track trends in contaminants over time (State of the Great Lakes 2009). Many of these programs have recently been expanded to include contaminants of emerging concern such as PBDEs and other flame retardants, perfluorochemicals (PFCs), and polychlorinated naphthalenes (PCNs).

In general, fish from Lakes Michigan, Ontario, and Huron have the highest levels of PCBs, DDT, and dieldrin; fish from Lake Superior have the highest levels of toxaphene; and those from Lake Ontario have the highest levels of mirex (State of the Great Lakes 2009). Contaminant levels are frequently lowest in fish from Lakes Superior and Erie and highest in Lakes Michigan and Ontario, presumably because of differences in agricultural, municipal, industrial, and airborne sources of contaminants. The higher toxaphene levels found in Lake Superior fish are an exception and due to the ability of the cold lake water to absorb this pesticide (Swackhamer et al. 1999). Mercury concentrations in fish are driven by several factors. Historically, chlor-alkali plants were point-source mercury discharge sources, but these plants have been closed. Modeling techniques, using source-receptor information, reveal that atmospheric mercury, mostly from coal-burning plants, is now the primary contributor to Great Lakes mercury concentrations (Cohen et al. 2004). Whether the conditions in local ecosystems are favorable to the methylation process further determines fish mercury concentration (Brown et al. 2010).

**Figure 1:**
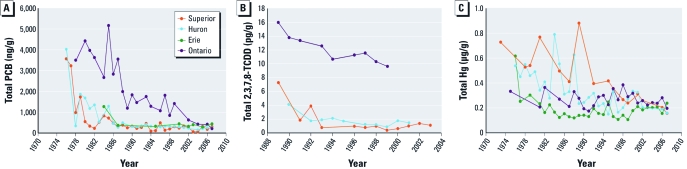
Contaminants in Great Lakes fish. (*A*) Total PCB in skinless fillets of 55- to 65-cm lake trout from the Canadian Great Lakes (adapted from Bhavsar et al. 2007). (*B*) Total 2,3,7,8-TCDD in skinless fillets of 60-cm lake trout from the Canadian Great Lakes (adapted from Bhavsar et al. 2008). (*C*) Total mercury in skinless fillets of 45- to 55-cm walleye from the Canadian Great Lakes (adapted from Bhavsar et al. 2010).

Substantial decreases in contaminant levels in Great Lakes top predator fish have been noted for many chemicals banned in the 1970s and 1980s, including PCBs, DDT, chlorodane, toxaphene, aldrin, dieldrin, and mirex (State of the Great Lakes 2009), although recent data suggest that contaminant levels are now decreasing more slowly or remaining level (Bhavsar et al. 2008, 2007; Carlson et al. 2010). As an example, PCB concentrations in lake trout have continuously declined since its manufacture ceased in the late 1970s, but the decline has slowed since the 1980s (Figure 1A) (Bhavsar et al. 2007; Carlson et al. 2010). The interlake differences, which were 2- to 9-fold in the 1970s and 1980s, are now only 2- to 4-fold (Bhavsar et al. 2007). Similarly, decreasing levels of 2,3,7,8-tetrachlorodibenzo-*p*-dioxin (2,3,7,8-TCDD) in lake trout from the Canadian Great Lakes have also been reported (Figure 1B) (Bhavsar et al. 2008). Mercury concentrations have generally declined over time, with some variation by fish species and lake (Figure 1C). From the 1970s to 2007, mercury levels varied 2- to 3-fold, with highest concentrations in Lake Superior and lowest in Lake Erie fish. However, as with other contaminants, spatial differences have diminished since 2000 (Bhavsar et al. 2010). Many factors may be affecting trends in contaminant levels, including reductions in external inputs and changes in the food web structure as a result of invasive species introduced during the late 1980s and early 1990s (Morrison et al. 1998).

Emerging contaminants of concern, including PBDEs and other persistent flame retardants, PFCs, and PCNs, have been measured in archived Great Lakes top predator fish to understand their temporal trends. PBDEs increased from the 1980s through the early 2000s in fish from all the Great Lakes (Zhu and Hites 2004), while more recently, congeners found in the discontinued penta and octa mixtures have started to decline in Lake Ontario fish (Ismail et al. 2009). A 4-fold increase in perfluoroctanesulfonate was found from 1980 to 2001 in Lake Ontario fish (Martin et al. 2004). PCNs have shown a dramatic (> 80%) decline since 1979; however, the levels (based on suggested dioxin toxic equivalency factors) are still above Ontario’s fish consumption advisory guidelines for dioxin-like compounds (Gewurtz et al. 2009).

*Contaminant exposures in Great Lakes fish consumers.* Human consumption of contaminated Great Lakes fish is a source of exposure to these pollutants. The duration and quantity of Great Lakes fish consumption is reflected in higher body burdens of PCBs, PCDD/Fs, persistent chlorinated pesticides, and mercury in studies of Great Lakes fish consumers (Cole et al. 2004; Falk et al. 1999; Fitzgerald et al. 1999; Hanrahan et al. 1999b; Kosatsky et al. 2000).

**Figure 2:**
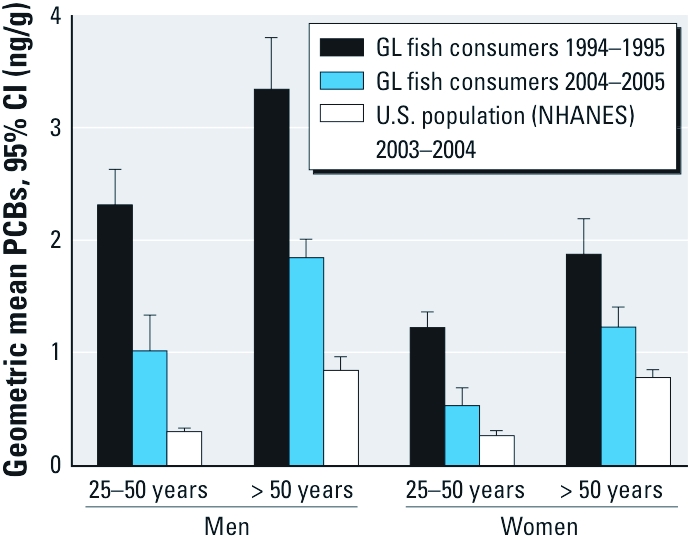
Sum of three PCB congeners in Great Lakes (GL) sport-fish consumers and a representative sample of the U.S. population. Great Lakes sport-fish consumers were from a cohort of licensed Great Lakes charter boat captains and their spouses. PCB levels were measured in serum samples from the cohort: 248 men and 189 women in 1994–1995 (PCBs 180, 153/132/105, and 138/163) and 262 men and 107 women in 2004–2005 (PCBs 180, 153/132, and 138/163) (Anderson et al. 2008a; Hanrahan et al. 1999b). NHANES measured PCBs in 331 men and 330 women in 2003–2004 (PCBs 180, 153/105, and 138) (Centers for Disease Control and Prevention 2006). Data are presented for NHANES participants with age and ethnicity similar to Great Lakes sport-fish consumers.

Figure 2 presents PCB levels in Great Lakes charter boat captains and their spouses, recruited because they consumed Great Lakes sport-caught fish, and levels in a nationally representative sample of the U.S. population of similar age and ethnicity [National Health and Nutrition Examination Survey (NHANES)]. PCB body burdens are higher in older age groups because of the higher exposures of this cohort born at the peak of PCB use and production (Quinn et al. 2011) as well slow metabolism and clearance, with some congeners having half-lives of ≥ 10 (O’Grady Milbrath et al. 2009). A temporal decline in PCBs was associated with decreased Great Lakes sport-caught fish consumption in this cohort (Figure 2) (Knobeloch et al. 2009), and a similar trend was also seen in another Great Lakes sport-caught fish consumer cohort (Tee et al. 2003). This decrease was also influenced by the decline in PCB levels in the fish as well as PCB levels in the diet overall (Diamond and Harrad 2009). Despite the declining levels, PCB levels remained higher in Great Lakes sport-caught fish consumers in 2004–2005 than in a representative sample of the U.S. population in 2003–2004 (Figure 2).

In this and other cohorts consuming Great Lakes sport-caught fish (Cole et al. 2002; Tee et al. 2003), men had higher serum levels of PCBs and persistent pesticides than women did. This may be related to lower fish intake by women, elimination of contaminants via pregnancy and lactation, and the fact that women in general have a higher percentage of body fat in which to store contaminants. However, Hanrahan et al. (1999a) found that although women ate less Great Lakes sport-caught fish than men, Great Lakes sport-caught fish consumption was a much stronger predictor of PCBs in women than length of lactation or adiposity. The transfer of maternal hydrophobic contaminants through the placenta and via breast milk is an important source of exposure for developing children (Lackmann et al. 1999; Quinn et al. 2011). PCB body burdens in adolescents who were breastfed were higher than those who were not breastfed (Schell and Gallo 2010). Variation in PCBs and other persistent pollutants across birth cohorts is a function of changes in both contaminant levels in fish and reproductive behaviors (Quinn et al. 2011).

*Health effects of contaminants.* Health effects of persistent pollutants have been seen in many studies, some with exposure levels similar to those found in Great Lakes fish consumers. Exposures during gestation, infancy, and early childhood pose the greatest risk, but other factors may also modify outcomes, including sex, genetics, health status, and nutritional status (Rice 2008).

Both PCBs and methylmercury are potent neurotoxicants, and the developing brain is considered more susceptible to impairment as a result of exposure than is the adult brain (Mergler et al. 2007; Rice 2008). Deficits in cognitive function, including effects on IQ, memory, verbal abilities, motor skills, and visual-spatial skills, have been seen with prenatal and early-life exposure (Boucher et al. 2009; Castoldi et al. 2008). Methylmercury is also known to increase the risk of cardiovascular disease and nervous system dysfunction (Guallar et al. 2002; Passos and Mergler 2008).

The most potent dioxin congener, 2,3,7,8-TCDD, has been identified as a human carcinogen, while the World Health Organization has rated PCBs and most chlorinated pesticides as probable human carcinogens (Steenland et al. 2004). PCBs, PCDD/Fs, and organochlorine pesticides have been associated with a variety of adverse health effects, and, in the populations consuming fish from the Great Lakes–St. Lawrence system, have been adversely associated with neurodevelopment and cognition in children (Jacobson et al. 1985, 1990; Lonky et al. 1996; Stewart et al. 2008) and adults (Haase et al. 2009; Schantz et al. 2001), *in utero* growth (Fein et al. 1984; Karmaus and Zhu 2004; Weisskopf et al. 2005), reproduction (Buck et al. 2000; Buck Louis et al. 2009; Courval et al. 1999), immune function (Schell and Gallo 2010), thyroid and steroid hormone regulation (Persky et al. 2001; Schell and Gallo 2010), diabetes (Codru et al. 2007; Gerstenberger et al. 2000; Turyk et al. 2009), and cardiovascular disease (Goncharov et al. 2008). However, fish consumption has not been associated with adverse health outcomes in all studies (e.g., Buck et al. 1999; Dellinger 2004).

## Omega-3 Fatty Acids and Great Lakes Fish

Fish are a good protein source with generally lower levels of saturated fats than other animal foods and are also substantial sources of selenium and omega-3 fatty acids (Institute of Medicine 2007). Omega-3 fatty acids are essential nutrients that are not synthesized by humans and thus must be consumed. The longer-chain omega-3 fatty acids, docosahexaenoic acid (DHA) and eicosapentaenoic acid (EPA), can be synthesized in humans from the shorter-chain α-linolenic acid, but the biosynthetic pathway is slow and inefficient (Surette 2008). DHA and EPA are synthesized by phytoplankton and bioconcentrate through the aquatic food chain. All seafood and aquatic organisms contain some of the longer-chain omega-3 fatty acids, but cold-water fatty fish contain more DHA and EPA than lean fish. Thus many of the fish species that have relatively high levels of omega-3 fatty acids also have relatively high levels of hydrophobic chemical pollutants.

Although there are considerable data on omega-3 fatty acids in numerous species of fish from marine and freshwater systems (Ackerman 2007), only three published reports are available, to our knowledge, on the content of omega-3 fatty acids in fish from the Great Lakes [Table 1; see also Supplemental Material, Table 1 (http://dx.doi.org/10.1289/ehp.1003396) for details on individual species]. Wang et al. (1990) studied Lake Superior fish fillets with skin on, Chan et al. (1999) examined skinned, defatted fish fillets collected from the St. Lawrence Seaway near Montreal, and Karahadian and Lindsay (1989) measured lipids extracted from skinned trout fillets from Lakes Superior and Michigan. Total median lipid in the Great Lakes fish varied, with higher levels in Lakes Superior and Michigan fish (8.5–9.0 g lipid/100 g fish) compared with the St. Lawrence River fish (1.4 g lipid/100 g fish). This difference may be related to variation in fish location, species, and fillet preparation methods. The sum of DHA + EPA accounted for 12–14% of total lipids in Great Lakes fish and 19–23% of total lipids in other types of fish, including marine fish purchased from Charleston (South Carolina) seafood markets (Gooch et al. 1987) and fatty, cold-temperature marine fish and commonly consumed types of seafood measured by the National Nutrient Database [U.S. Department of Agriculture (USDA) 2010] (Table 1). Median DHA + EPA levels are, respectively, 1,048 and 1,062 mg/100 g fish for two studies of Great Lakes fish, 214 mg/100 g fish for marine fish from North Carolina, 263 mg/100 g fish for commonly consumed fish, and 1,449 mg/100 g fish for fatty, cold-temperature marine fish. However, direct comparison of total DHA + EPA/100 g fish in these studies may not be appropriate because the methodology for fish sample preparation varied by study. More data are needed to evaluate levels of omega-3 fatty acids, mercury, and POPs in commonly consumed fish from all of the Great Lakes, before and after cooking. For comparison purposes, it is critical that techniques for preparing the fillets for testing be standardized across studies.

**Table 1:** Omega-3 fatty acids in Great Lakes and commercial fish: summary data from multiple species are described in Supplemental Material, Table 1 (http://dx.doi.org/10.1289/ehp.1003396).

Fish source	Sample	*n*	Descriptive value	Total fat g/100 g fish	Omega-3 g/100 g lipid	Omega-3 mg/100 g fish	DHA + EPA g/100 g lipid	DHA + EPA mg/100 g fish	Reference
Lake Superior		Fillet with skin		86		Median		8.5		29.0		2,611		13.5		1,062		Wang et al. 1990
						Minimum		1.0		24.1		260		10.2		180		
						Maximum		25.7		37.6		6,194		18.0		3,033		
St. Lawrence River		Fillet skinned defatted		57		Median		1.4		21.3		281		—		—		Chan et al. 1999
						Minimum		1.0		5.7		63		—		—		
						Maximum		3.3		29.1		818		—		—		
Lakes Superior and Michigan		Oil from fillet skinned		12		Median		9.0		20.5		1,937		12.2		1,048		Karahadian and Lindsay 1989
						Minimum		4.0		18.2		832		10.4		536		
						Maximum		36.0		23.6		6,552		13.6		3,960		
Southeastern United States (Charleston, SC, seafood market)		Fillet skinned		87		Median		1.2		—		—		18.8		214		Gooch et al. 1987
						Minimum		0.6		—		—		8.2		55		
						Maximum		14.6		—		—		34.6		1,393		
Commonly consumed seafood in United States*a*		Edible portion		—		Median		1.0		—		—		22.7		263		USDA 2010
						Minimum		0.7		—		—		1.2		33		
						Maximum		6.3		—		—		43.0		1,436		
Fatty, cold-temperature marine fish		Edible portion		—		Median		6.3		—		—		22.6		1,449		USDA 2010
						Minimum		4.8		—		—		16.5		1,173		
						Maximum		13.9		—		—		30.2		2,299		
Abbreviations: DHA, docosahexaenoic acid; EPA, eicosapentaenoic acid. **a**Types of fish frequently consumed in the United States [see Supplemental Material, Table 1 (http://dx.doi.org/10.1289/ehp.1003396)] as identified by the National Health and Nutrition Examination Survey (NHANES) (Mahaffey et al. 2008) and the National Marine Fisheries Service (National Fisheries Institute Inc. 2008).																		

*Omega-3 fatty acids in Great Lakes fish consumers.* Consumption of fish, and fatty fish in particular, is correlated with serum levels of omega-3 fatty acids. For example, both fish and fish oil consumption were related to plasma DHA and EPA in a cohort of 4,949 men and women from the United Kingdom (Welch et al. 2006). However, the relationship of Great Lakes fish consumption to omega-3 fatty-acid levels in serum is unclear. A study that included 243 consumers of sport fish from the St. Lawrence River concluded that neither total fish nor locally caught fish intake was related to serum omega-3 fatty acids, while a positive association was found between serum omega-3 fatty acids and commercial fatty-fish intake (Philibert et al. 2006). Similarly, a second study of 112 male St. Lawrence River fish consumers found no association between fish intake and serum omega-3 fatty acids (Godin et al. 2003). A third study recruited 86 Asian and Euro-Canadians who consumed at least 26 meals of Great Lakes fish annually (Cole et al. 2002). For Euro-Canadians, total plasma DHA and EPA were associated with total fish meals and with inland fish meals, but not with Great Lakes fish meals. The lack of an association between Great Lakes fish intake and serum omega-3 fatty acids in these studies may be related to small sample sizes, at least in the second and third studies, or associations may have been obscured by fatty-acid intake from other unmeasured sources or imprecise measurement of fish intake. Furthermore, participants may have been consuming fish low in total fat and/or omega-3 fatty acid (Godin et al. 2003), but none of these studies measured levels of omega-3 fatty acids in the fish actually consumed by the participants. The absence of a relationship between freshwater fish consumption and serum omega-3 fatty-acid levels in these three studies of moderate and high fish consumers needs to be confirmed in other investigations that measure omega-3 fatty acids in both fish consumers and in the fish they consume and should be expanded to include more of the fish-eating populations around the Great Lakes.

*Health effects of omega-3 fatty acids.* Omega-3 fatty acids are major components of neuronal, retinal, and myocardial membranes. DHA constitutes 40% of all omega-3 fatty acids in the brain and is important for neurological development and function (Dyall and Michael-Titus 2008). Consumption during pregnancy of fish not contaminated with mercury has been associated with increased cognitive function in the offspring (Daniels et al. 2004; Hibbeln et al. 2007), although the assessment of mercury and its adverse health effects were suboptimal in these studies (Mahaffey and Schoeny 2007). Fish consumption has been linked with increased cognitive performance measured 3 years later in male adolescents compared with males who ate less fish (Å^^´^^berg et al. 2009). Oken et al. (2005) found that whereas higher fish consumption during pregnancy was associated with improved infant cognition, higher maternal hair mercury was associated with lower cognition, highlighting the dilemma for fish consumers.

Substantial evidence suggests that increased fish consumption has important cardiovascular benefits (Bushkin-Bedient and Carpenter 2010). Several randomized controlled trials indicated that fish consumption and/or omega-3 supplementation reduced the risk of sudden death in men recovering from a heart attack (Burr et al. 1989; Marchioli et al. 2002; Yokoyama et al. 2007). The mechanism proposed is that omega-3 fatty acids increase membrane fluidity of cardiac muscle, reducing the risk of arrhythmia (Leaf et al. 2008). However, it is not clear that fish consumption reduces the risk of development of an initial heart attack (Burr et al. 2005; Yokoyama et al. 2007). Beneficial effects on the cardiovascular and nervous systems from fish consumption are thought to come primarily from the omega-3 fatty acids, but may also be related to decreased consumption of other animal fats (Bushkin-Bedient and Carpenter 2010).

## Consumption of Great Lakes Fish

Consumption of Great Lakes fish is of particular concern for several population groups. Consumption rates in women and children are important because of known developmental effects of pollutants. In addition, potentially larger pollutant exposures are possible in persons exposed through recreational, cultural or subsistence fishing. Because all fish carry some levels of pollutants, it is necessary to consider total fish consumption rates, including commercial and sport-caught fish from the Great Lakes and other bodies of water.

In surveys of Great Lakes states populations, 7–8% of adults, or 4.2–4.7 million persons, reported consumption of Great Lakes sport-caught fish in the previous year, and 830,000 persons ate ≥ 24 Great Lakes sport-caught fish meals annually (Imm et al. 2005; Tilden et al. 1997). Adults who included Great Lakes sport-caught fish in their diet consumed more fish than those who consumed only commercially purchased fish. Great Lakes sport-caught fish consumption rates were higher in men than women. Fish consumption rates for persons consuming Great Lakes sport-caught fish in these two surveys are compared with consumption rates reported in studies of Great Lakes recreational and native anglers in Table 2. Maximum annual Great Lakes fish meals in these studies ranged from 126 to 960, suggesting large exposures to contaminants in some Great Lakes sport-caught fish consumers, depending on the types of fish consumed. Commercial Great Lakes fish are sold widely in both Canada and the United States, but information about consumption rates is lacking.

**Table 2:** Total and Great Lakes fish consumption in populations consuming Great Lakes fish.

Study group	*n*	Location	Date	Mean total fish meals/year	Mean GL sport-fish meals/year	Maximum annual GL sport-fish meals	Reference
GL sport-fish consumers*a*		679		GL states		1993–1994		48		7*b*		292		Tilden et al. 1997
GL sport-fish consumers*a*		299		GL states		2001–2002		53		13		126		Imm et al. 2005
Anglers		2,542		Wisconsin, Illinois, Indiana, Michigan, Ohio		1994–1995		42–53		28–47		365		Hanrahan et al. 1999b
Anglers		117		Lake St. Pierre on St. Lawrence River		2003		83*c*		34*c*		960		Abdelouahab et al. 2008
Anglers		112		St. Lawrence River		1996		89–111		55–77		—		Godin et al. 2003
Anglers		113		St. Lawrence River*d*		1992		70–81		28–37		245		Kearney and Cole 2003
Mohawks		139		St. Lawrence River		1992–1995		—		21		—		Fitzgerald et al. 1999
Mohawks		22		St. Lawrence River		1996		—		53*c*		—		Chan et al. 1999
Ojibwe		271		Lakes Michigan, Huron, Superior		1993–2000		95*e*		—		—		Dellinger 2004
Ojibwe		346		Lake Superior		1993–2000		104*e*		—		—		Dellinger 2004
GL, Great Lakes. **a**Data are from the 7–8% of persons who reported consuming Great Lakes fish in random survey of Great Lakes states residents. **b**Median fish meals per year. **c**Converted from grams per day to meals per year based on average fish meal size of 227 g per 70-kg body weight (formula from Anderson et al. 1993). **d**Anglers from Cornwall, Ontario, who fished predominantly on St. Lawrence River. **e**Predominantly Great Lakes fish meals (personal communication, J. Dellinger).														

Consumption of sport-caught fish in children living in the Great Lakes Basin is related to their parents’ sport-fish consumption (Broussard and Haley 2005; Imm et al. 2007; Michigan Department of Community Health 2007). A survey of the general population in Wisconsin and Minnesota found that 29–39% of children 2–17 years of age ate sport-caught fish, although consumption was infrequent, with more than half of the children eating less than six meals of sport-caught fish annually (Imm et al. 2007). A study of children of New York anglers found that 39–48% of 4- to 10-year-old children consumed sport-caught fish (median 2–3 annual meals, range 49 meals) and 15–18% consumed Lake Ontario sport-caught fish (median 1.5–2 annual meals, range up to 29 meals) (Beehler et al. 2002). Consumption rates were lower for younger children: 5% of 1-year-old, 22% of 2-year-old, and 35% of 3-year-old children of anglers ate sport-caught fish. These data suggest that children may be exposed to toxicants from Great Lakes fish during important developmental stages. However, because *in utero* toxicant exposures may pose a greater risk for adverse neurodevelopmental and other health outcomes than exposure after birth (Mergler et al. 2007; Rice 2008), Great Lakes fish consumption in women of childbearing age is also a concern. Table 3 summarizes data on total fish and sport-caught fish consumption in studies of all adults, reproductive-age women, and pregnant women from the Great Lakes Basin. In general, the proportions of persons consuming any fish and sport-caught fish were similar in these three groups. Maximum rates indicated that some pregnant women greatly exceeded two fish meals per week. However, overall fish and shellfish consumption in reproductive-age women from the coastal regions of the Great Lakes states is lower compared with other coastal regions of the United States (Mahaffey et al. 2009).

**Table 3:** Fish consumption in adults, women of reproductive age, and pregnant women in the Great Lakes Basin.

Population	Location	Date	*n*	Percentage eating any fish	Mean total fish meals/year	Maximum total fish meals/month	Percentage eating sport fish	Mean GL sport-fish meals/year	Maximum GL sport-fish meals/month	Reference
Adults*a*		GL states		1993–1994		8,078		88		29*b*		—		27		7*b*		24		Tilden et al. 1997
Adults*a*		GL states		2001–2002		4,054		84		38		—		22		13		11		Imm et al. 2005
Females 16–49 years of age*a*		GL states		1999–2004		1,280		76*c*		42–47*d*		—		—		—		—		Mahaffey et al. 2009
Females 18–45 years of age*a*		Wisconsin		1998–1999		596		90		—		—		30		—		—		Anderson et al. 2004
Pregnant		Ontario		2002–2005		2,394		68*e*		88		—		—		—		—		Sontrop et al. 2007
Pregnant		Wisconsin		2003		726		85		36		60		29		—		—		Gliori et al. 2006
Prepregnancy		Quebec		1999–2001		159		89		43		19.5		27		6		8.5		Morrissette et al. 2004
Pregnant		Quebec		1999–2001		159		83		38		31.5		22		4		4.5		Morrissette et al. 2004
Pregnant		Illinois		1994–1996		484		90		—		—		10*f*		—		—		Waller et al. 1996
GL, Great Lakes. **a**Population-based survey. **b**Median fish meals/year. **c**Consumed more than rarely. **d**Estimated from Figure 3. **e**Consumed fish ≥ 1 time/week. **f**Consumed regularly.																				

## Risk–Benefit Analysis of Great Lakes Fish Consumption and Additional Needs

Despite the need for such assessments, there is currently no accepted method to compare net risks with net benefits of consuming fish (Domingo et al. 2007; Ginsberg and Toal 2009; Guevel et al. 2008; van der Voet et al. 2007). The few risk–benefit assessments for fish consumption that have been attempted have focused on methylmercury and omega-3 fatty acids in relation to cardiovascular disease and/or neurobehavioral outcomes (Guallar et al. 2002; Mahaffey et al. 2008). Overall health risks related to PCB and PCDD/F exposure through farmed salmon consumption have also been assessed (Hites et al. 2004). Some investigators have suggested that cardiovascular benefits from consumption of farmed salmon for adults > 25 years of age outweigh cancer risks by 100-fold or more (Mozaffarian and Rimm 2006), while another group has calculated that the cumulative cancer risk from consumption of farmed salmon at rates that provide 1 g/day DHA + EPA would be 24 times higher than acceptable risk levels (Foran et al. 2005). Risks related to PCBs, PCDD/Fs, and persistent pesticides are especially critical to evaluate for Great Lakes fish consumers because concentrations of these chemicals are in general higher than in commercial fish, as reflected by higher contaminant body burdens in Great Lakes fish consumers than in commercial fish consumers (Hanrahan et al. 1999b). On the other hand, based on contamination levels, risks from mercury exposure may be less in Great Lakes fish consumers than in marine fish consumers. In a study that followed Quebec women during pregnancy, the contribution of commercial fish (especially canned tuna) to mercury exposure was found to be more important than that from sport fish from the St. Lawrence River (Morrissette et al. 2004). Few, if any, studies have evaluated cumulative risk from both PCBs and mercury. This is an important question that needs to be addressed, as most Great Lakes fish consumers also consume commercially available marine fish (Hanrahan et al. 1999b; Imm et al. 2005).

In adults, risk assessments for fish frequently have focused on cardiovascular disease and mortality. However, given the body of evidence for other health effects related to hydrophobic toxicants in Great Lakes fish, other end points such as cancer, diabetes, reproductive outcomes, immune system dysfunction, and decreased cognitive performance should be considered (e.g., Schantz et al. 2001; Schell and Gallo 2010; Turyk et al. 2009; Weisskopf et al. 2005). Although fish consumption has been reported to protect against cardiovascular disease (e.g., Daviglus et al. 1997; Hu et al. 2002), studies of Great Lakes fish consumers did not find protective associations of fish intake with cardiovascular disease mortality (Tomasallo et al. 2010) or risk factors for cardiovascular disease (Godin et al. 2003).

Ideally, risk assessments for Great Lakes fish should also consider factors that may interact with fish contaminants to affect health end points such as neurodevelopment (Castoldi et al. 2008; Mergler et al. 2007). These factors include *a*) coexposures to multiple toxic pollutants from fish and other sources, including methylmercury, lead, and POPs; *b*) dietary intakes of nutrients such as omega-3 fatty acids, selenium, and other antioxidants—either from fish or from other sources—that could possibly counteract the negative effects of contaminants; *c*) sex differences in exposure or susceptibility; and *d*) the social environment. An investigation of the neurodevelopmental impact of fish consumed from all sources found that simultaneous adjustment for both methylmercury and fish intake strengthened estimates of both benefits related to fish consumption and risks of mercury exposure (Oken et al. 2008). Thus, not controlling for beneficial factors can result in misestimation of health risks of toxic contaminants (Budtz-Jorgensen et al. 2007).

Other factors to consider in risk assessment include differences in Great Lakes fish contaminants by species, size, and location. Meal size and storage and preparation methods, which can reduce exposure to lipophilic contaminants and/or degrade fatty acids (Moses et al. 2009), may vary for native populations, immigrant groups such as the Hmong, low-income persons who fish for subsistence, and recreational anglers. In native, immigrant, and subsistence populations, sport-caught fish may be a diet staple that is difficult to replace because of economic constraints or unavailability of alternate food in isolated locales or because it would be replaced by less-nutritious foods with a less desirable fat profile, either way resulting in decreased health status [Dellinger 2004; Donaldson et al. 2010; U.S. EPA and Toxicology Excellence for Risk Assessment (TERA) 1999]. Furthermore, fishing may contribute to social, cultural, and recreational activities that have other health benefits. As a cultural resource, fishing may hold a prominent place in religious and social ceremonies, teach survival skills, and contribute to social bonding in the family and community (Donaldson et al. 2010; Wheatley and Wheatley 2000). However, few studies (Beehler et al. 2003; DeWeese et al. 2009) have documented the social and cultural benefits of fishing in populations consuming Great Lakes fish.

In conclusion, extensive data from many studies are available on the concentrations of bioaccumulative contaminants, including PCBs and DDE, both in Great Lakes fish and in Great Lakes fish consumers. In contrast, studies on Great Lakes fish consumers that include concurrent measures of omega-3 fatty acids, chemical contaminants, and health end points are lacking. Such studies would provide the strongest data to address the question as to whether the benefits of Great Lakes fish consumption outweigh the risks posed by ingestion of contaminants in the fish.

In the absence of such studies, the most pressing need is for data on the omega-3 fatty-acid content in various fish species in the five Great Lakes. What little information has been published on the omega-3 fatty-acid content of even a limited number of fish species from Lake Superior, Lake Michigan, and the St. Lawrence River is > 10 years old. Moreover, the different species of fish in the five Great Lakes would be expected to vary in fatty-acid content depending on characteristics such as size, overall fat content, and location and temperature of the waters where they were caught. What sparce data exist on omega-3 fatty-acid levels in consumers of fish from the Great Lakes-St. Lawrence system do not suggest a correlation between serum fatty-acid concentrations and consumption of fish from these waters. Further, none of the available studies actually measured the fatty-acid content of the fish being consumed.

Thus, our knowledge has critical gaps that need to be filled before a practical risk–benefit analysis can be made for the human consumption of Great Lakes fish. We hope that this review will spur critical research that will fill these data gaps and permit risk–benefit analysis of Great Lakes fish consumption.

## Supplemental Material

(115 KB) PDF

## References

[r1] Abdelouahab N, Vanier C, Baldwin M, Garceau S, Lucotte M, Mergler D. (2008). Ecosystem matters: fish consumption, mercury intake and exposure among fluvial lake fish-eaters.. Sci Total Environ.

[r2] Å^^’^^berg MA, Å^^’^^berg N, Brisman J, Sundberg R, Winkvist A, Torén K (2009).

[r3] Ackerman RG (2007). Fatty acids in fish and shellfish.

[r4] American Heart Association (2010). Fish and Omega-3 Fatty Acids.. http://www.americanheart.org/presenter.jhtml?identifier=4632.

[r5] Anderson H, Amrhein JF, Shubat P, Hesse J (1993). Protocol for a Uniform Great Lakes Sport Fish Consumption Advisory.. http://fn.cfs.purdue.edu/fish4health/HealthRisks/TaskForce.pdf.

[r6] Anderson H, McCann P, Stahl J, LaNetta A, Hornshaw T, Day R, et al (2007). A Protocol for Mercury-based Fish Consumption Advice.. http://dhs.wi.gov/eh/fish/FishFS/2007Hg_Add_Final_05_07.pdf.

[r7] Anderson HA, Hanrahan LP, Smith A, Draheim L, Kanarek M, Olsen J (2004). The role of sport-fish consumption advisories in mercury risk communication: a 1998–1999 12-state survey of women age 18–45.. Environ Res.

[r8] Anderson HA, Imm P, Knobeloch L, Turyk M, Mathew J, Buelow C (2008a). Polybrominated diphenyl ethers (PBDE) in serum: findings from a US cohort of consumers of sport-caught fish.. Chemosphere.

[r9] Anderson S Allen P Peckham S Goodwin N. 2008b Asking the right questions: scoping studies in the commissioning of research on the organization and delivery of health services. Health Res Policy Syst; doi:10.1186/1478-4505-6-7 [Online 9 July 2008] PMC2500008 18613961

[r10] Ashizawa AE, Hicks HE, De Rosa CT (2005). Human health research and policy development: experience in the Great Lakes region.. Int J Hyg Environ Health.

[r11] Beehler GP, McGuinness BM, Vena JE (2003). Characterizing Latino anglers’ environmental risk perceptions, sport fish consumption, and advisory awareness.. Med Anthropol Q.

[r12] Beehler GP, Weiner JM, McCann SE, Vena JE, Sandberg DE (2002). Identification of sport fish consumption patterns in families of recreational anglers through factor analysis.. Environ Res.

[r13] Bhavsar SP, Awad E, Fletcher R, Hayton A, Somers KM, Kolic T (2008). Temporal trends and spatial distribution of dioxins and furans in lake trout or lake whitefish from the Canadian Great Lakes.. Chemosphere.

[r14] Bhavsar SP Awad E Mahon CG Petro S 2011 Great Lakes fish consumption advisories: is mercury a concern? Ecotoxicology; doi:10.1007/s10646-0731-0 [Online 12 July 2011] 21748390

[r15] Bhavsar SP, Gewurtz SB, McGoldrick DJ, Keir MJ, Backus SM (2010). Changes in mercury levels in Great Lakes fish between 1970s and 2007.. Environ Sci Technol.

[r16] Bhavsar SP, Jackson DA, Hayton A, Reiner EJ, Chen T, Bodner J (2007). Are PCB levels in fish from the Canadian Great Lakes still declining?. J Great Lakes Res.

[r17] Boucher O, Muckle G, Bastien CH (2009). Prenatal exposure to polychlorinated biphenyls: a neuropsychologic analysis.. Environ Health Perspect.

[r18] Broussard S, Haley A (2005). 2005 Indiana Licensed Angler Survey.. http://fn.cfs.purdue.edu/fish4health/HealthRisks/AnglerSurvey05.pdf.

[r19] Brown D, Goncharov A, Paul E, Simonin H, Carpenter DO (2010). The relationship between Adirondack Lake pH and levels of mercury in yellow perch.. J Aquat Anim Health.

[r20] Buck GM, Mendola P, Vena JE, Sever LE, Kostyniak P, Greizerstein H (1999). Paternal Lake Ontario fish consumption and risk of conception delay, New York State Angler Cohort.. Environ Res.

[r21] Buck GM, Vena JE, Schisterman EF, Dmochowski J, Mendola P, Sever LE (2000). Parental consumption of contaminated sport fish from Lake Ontario and predicted fecundability.. Epidemiology.

[r22] Buck Louis GM, Dmochowski J, Lynch C, Kostyniak P, McGuinness BM, Vena JE (2009). Polychlorinated biphenyl serum concentrations, lifestyle and time-to-pregnancy.. Hum Reprod.

[r23] Budtz-Jorgensen E, Grandjean P, Weihe P. (2007). Separation of risks and benefits of seafood intake.. Environ Health Perspect.

[r24] Burr ML, Dunstan FD, George CH (2005). Is fish oil good or bad for heart disease? Two trials with apparently conflicting results.. J Membr Biol.

[r25] Burr ML, Fehily AM, Gilbert JF, Rogers S, Holliday RM, Sweetnam PM (1989). Effects of changes in fat, fish, and fibre intakes on death and myocardial reinfarction: diet and reinfarction trial (DART).. Lancet.

[r26] Bushkin-Bedient S, Carpenter DO (2010). Benefits versus risks associated with consumption of fish and other seafood.. Rev Environ Health.

[r27] Carlson DL, Vault DS, Swackhamer DL (2010). On the rate of decline of persistent organic contaminants in lake trout (*Salvelinus namaycush*) from the Great Lakes, 1970–2003.. Environ Sci Technol.

[r28] Castoldi AF, Johansson C, Onishchenko N, Coccini T, Roda E, Vahter M (2008). Human developmental neurotoxicity of methylmercury: impact of variables and risk modifiers.. Regul Toxicol Pharmacol.

[r29] Centers for Disease Control and Prevention (2006). Laboratory Procedure Manual: PCBs and Persistent Pesticides.. http://www.cdc.gov/nchs/data/nhanes/nhanes_03_04/l28_c_met_%20PCBs_and_Persistent_Pesticides.pdf.

[r30] Chan HM, Trifonopoulos M, Ing A, Receveur O, Johnson E (1999). Consumption of freshwater fish in Kahnawake: risks and benefits.. Environ Res.

[r31] Codru N, Schymura MJ, Negoita S, Rej R, Carpenter DO (2007). Diabetes in relation to serum levels of polychlorinated biphenyls and chlorinated pesticides in adult Native Americans.. Environ Health Perspect.

[r32] Cohen M, Artz R, Draxler R, Miller P, Poissant L, Niemi D (2004). Modeling the atmospheric transport and deposition of mercury to the Great Lakes.. Environ Res.

[r33] Cole DC, Kearney J, Sanin LH, Leblanc A, Weber JP (2004). Blood mercury levels among Ontario anglers and sport-fish eaters.. Environ Res.

[r34] Cole DC, Sheeshka J, Murkin EJ, Kearney J, Scott F, Ferron LA (2002). Dietary intakes and plasma organochlorine contaminant levels among Great Lakes fish eaters.. Arch Environ Health.

[r35] Courval JM, DeHoog JV, Stein AD, Tay EM, He J, Humphrey HE (1999). Sport-caught fish consumption and conception delay in licensed Michigan anglers.. Environ Res.

[r36] Daniels JL, Longnecker MP, Rowland AS, Golding J, The ALSPAC Study Team–University of Bristol Institute of Child Health. (2004). Fish intake during pregnancy and early cognitive development of offspring.. Epidemiology.

[r37] Daviglus ML, Stamler J, Orencia AJ, Dyer AR, Liu K, Greenland P (1997). Fish consumption and the 30-year risk of fatal myocardial infarction.. New Engl J Med.

[r38] Dellinger JA (2004). Exposure assessment and initial intervention regarding fish consumption of tribal members of the Upper Great Lakes Region in the United States.. Environ Res.

[r39] DeWeese AD, Kmiecik NE, Chiriboga ED, Foran JA (2009). Efficacy of risk-based, culturally sensitive Ogaa (walleye) consumption advice for Anishinaabe tribal members in the Great Lakes region.. Risk Anal.

[r40] Diamond M, Harrad S (2009). The chemicals that will not go away: implications for human exposures to reservoirs of POPs. In: Persistent Organic Pollutants: Current Issues and Future Challenges (Harrad SJ, ed).

[r41] Domingo JL, Bocio A, Marti-Cid R, Llobet JM (2007). Benefits and risks of fish consumption. Part II. RIBEPEIX, a computer program to optimize the balance between the intake of omega-3 fatty acids and chemical contaminants.. Toxicology.

[r42] Donaldson SG, Van Osstdam J, Tikhonov C, Feeley M, Armstrong B, Ayotte P (2010). Environmental contaminants and human health in the Canadian Arctic.. Sci Total Environ.

[r43] Dyall SC, Michael-Titus AT (2008). Neurological benefits of omega-3 fatty acids.. Neuromolecular Med.

[r44] Falk C, Hanrahan L, Anderson HA, Kanarek MS, Draheim L, Needham L (1999). Body burden levels of dioxin, furans, and PCBs among frequent consumers of Great Lakes sport fish. The Great Lakes Consortium.. Environ Res.

[r45] Fein GG, Jacobson JL, Jacobson SW, Schwartz PM, Dowler JK (1984). Prenatal exposure to polychlorinated biphenyls: effects on birth size and gestational age.. J Pediatr.

[r46] Fitzgerald EF, Deres DA, Hwang SA, Bush B, Yang BZ, Tarbell A (1999). Local fish consumption and serum PCB concentrations among Mohawk men at Akwesasne.. Environ Res.

[r47] Foran JA, Good DH, Carpenter DO, Hamilton MC, Knuth BA, Schwager SJ (2005). Quantitative analysis of the benefits and risks of consuming farmed and wild salmon.. J Nutr.

[r48] Gerstenberger SL, Dellinger JA, Hansen LG (2000). Concentrations and frequencies of polychlorinated biphenyl congeners in a Native American population that consumes Great Lakes fish.. J Toxicol Clin Toxicol.

[r49] Gewurtz SB, Lega R, Crozier PW, Whittle DM, Fayez L, Reiner EJ (2009). Factors influencing trends of polychlorinated naphthalenes and other dioxin-like compounds in lake trout (*Salvelinus namaycush*) from Lake Ontario, North America (1979–2004).. Environ Toxicol Chem.

[r50] Ginsberg GL, Toal BF (2009). Quantitative approach for incorporating methylmercury risks and omega-3 fatty acid benefits in developing species-specific fish consumption advice.. Environ Health Perspect.

[r51] Gliori G, Imm P, Anderson HA, Knobeloch L (2006). Fish consumption and advisory awareness among expectant women.. WMJ.

[r52] Godin C, Shatenstein B, Paradis G, Kosatsky T. (2003). Absence of cardiovascular benefits and sportfish consumption among St. Lawrence River anglers.. Environ Res.

[r53] Goncharov A, Haase RF, Santiago-Rivera A, Morse G, McCaffrey RJ, Rej R (2008). High serum PCBs are associated with elevation of serum lipids and cardiovascular disease in a Native American population.. Environ Res.

[r54] Gooch JA, Hale MB, Brown TJ, Bonnet JC, Brand CG, Regier LW (1987).

[r55] Great Lakes Information Network (2010). Great Lakes Fish Consumption Advisories.. http://www.great-lakes.net/envt/flora-fauna/wildlife/fishadv.html#state.

[r56] Guallar E, Sanz-Gallardo MI, van’t Veer P, Bode P, Aro A, Gomez-Aracena J (2002). Mercury, fish oils, and the risk of myocardial infarction.. N Engl J Med.

[r57] Guevel MR, Sirot V, Volatier JL, Leblanc JC (2008). A risk-benefit analysis of French high fish consumption: a QALY approach.. Risk Anal.

[r58] Haase RF, McCaffrey RJ, Santiago-Rivera AL, Morse GS, Tarbell A (2009). Evidence of an age-related threshold effect of polychlorinated biphenyls (PCBs) on neuropsychological functioning in a Native American population.. Environ Res.

[r59] Hanrahan LP, Anderson HA, Falk C, Olson J (1999a). Reproductive predictors of serum PCB and DDE levels among frequent Great Lakes sport fish consumers: the role of gender, births, and breastfeeding.. Eur J Oncol.

[r60] Hanrahan LP, Falk C, Anderson HA, Draheim L, Kanarek MS, Olson J (1999b). Serum PCB and DDE levels of frequent Great Lakes sport fish consumers—a first look.. Environ Res.

[r61] Health Canada (2002). Consumption Advice: Making Informed Choices about Fish.. http://www.hc-sc.gc.ca/fn-an/securit/chem-chim/environ/mercur/cons-adv-etud-eng.php.

[r62] Hibbeln JR, Davis JM, Steer C, Emmett P, Rogers I, Williams C, Golding J (2007). Maternal seafood consumption in pregnancy and neurodevelopmental outcomes in childhood (ALSPAC study): an observational cohort study.. Lancet.

[r63] Hites RA, Foran JA, Carpenter DO, Hamilton MC, Knuth BA, Schwager SJ (2004). Global assessment of organic contaminants in farmed salmon.. Science.

[r64] Hu FB, Bronner L, Willett WC, Stampfer MJ, Rexrode KM, Albert CM (2002). Fish and omega-3 fatty acid intake and risk of coronary heart disease in women.. JAMA.

[r65] Imm P, Knobeloch L, Anderson HA (2005). Fish consumption and advisory awareness in the Great Lakes Basin.. Environ Health Perspect.

[r66] Imm P, Knobeloch L, Anderson HA (2007). Maternal recall of children’s consumption of commercial and sport-caught fish: findings from a multi-state study.. Environ Res.

[r67] Institute of Medicine (2007). Seafood Choices: Balancing Benefits and Risks.

[r68] Ismail N, Gewurtz SB, Pleskach K, Whittle DM, Helm PA, Marvin CH (2009). Brominated and chlorinated flame retardants in Lake Ontario, Canada, lake trout (*Salvelinus namaycush*) between 1979 and 2004 and possible influences of food-web changes.. Environ Toxicol Chem.

[r69] Jacobson JL, Jacobson SW, Humphrey HE (1990). Effects of *in utero* exposure to polychlorinated biphenyls and related contaminants on cognitive functioning in young children.. J Pediatr.

[r70] Jacobson SW, Fein GG, Jacobson JL, Schwartz PM, Dowler JK (1985). The effect of intrauterine PCB exposure on visual recognition memory.. Child Dev.

[r71] Karahadian C, Lindsay RC (1989). Composition of n-3 oils from some Great Lakes freshwater fish.. J Food Compost Anal.

[r72] Karmaus W Zhu X. 2004 Maternal concentration of polychlorinated biphenyls and dichlorodiphenyl dichlorethylene and birth weight in Michigan fish eaters: a cohort study. Environ Health 3 1; doi:10.1186/1476-069X-3-1 [Online 28 January 2004] 14748928 PMC356928

[r73] Kearney JP, Cole DC (2003). Great Lakes and inland sport fish consumption by licensed anglers in two Ontario communities.. J Great Lakes Res.

[r74] Kinnunen RW (2003). Great Lakes commercial fisheries.. http://www.miseagrant.umich.edu/downloads/fisheries/GLCommercialFinal.pdf.

[r75] Knobeloch L, Turyk M, Imm P, Schrank C, Anderson H. (2009). Temporal changes in PCB and DDE levels among a cohort of frequent and infrequent consumers of Great Lakes sportfish.. Environ Res.

[r76] Kosatsky T, Przybysz R, Armstrong B. (2000). Mercury exposure in Montrealers who eat St. Lawrence River sportfish.. Environ Res.

[r77] Kris-Etherton PM Harris WS Appel LJ, for theNutrition Committee. 2003 Fish consumption, fish oil, omega-3 fatty acids, and cardiovascular disease. AHA Scientific Statement. Arterioscler Thromb Vasc Biol 23 e20 e30 12588785 10.1161/01.atv.0000038493.65177.94

[r78] Lackmann GM, Angerer J, Salzberger U, Tollner U (1999). Influence of maternal age and duration of pregnancy on serum concentrations of polychlorinated biphenyls and hexachlorobenzene in full-term neonates.. Biol Neonate.

[r79] Leaf A, Kang JX, Xiao YF (2008). Fish oil fatty acids as cardiovascular drugs.. Curr Vasc Pharmacol.

[r80] Li G, Sinclair AJ, Li D (2011). Comparison of lipid content and fatty acid composition in the edible meat of wild and cultured freshwater and marine fish and shrimps from China.. J Agric Food Chem.

[r81] Lonky E, Reihman J, Darvill T, Mather JJ, Daly H (1996). Neonatal behavioral assessment scale performance in humans influenced by maternal consumption of environmentally contaminated Lake Ontario fish.. J Great Lakes Res.

[r82] Mahaffey KR, Clickner RP, Jeffries RA (2008). Methylmercury and omega-3 fatty acids: co-occurrence of dietary sources with emphasis on fish and shellfish.. Environ Res.

[r83] Mahaffey KR, Clickner RP, Jeffries RA (2009). Adult women’s blood mercury concentrations vary regionally in USA: association with patterns of fish consumption (NHANES 1999–2004).. Environ Health Perspect.

[r84] Mahaffey KR, Schoeny R (2007). Maternal seafood consumption and children’s development.. Lancet.

[r85] Marchioli R, Barzi F, Bomba E, Chieffo C, Di Gregorio D, Di Mascio R (2002). Early protection against sudden death by n-3 polyunsaturated fatty acids after myocardial infarction: time-course analysis of the results of the Gruppo Italiano per lo Studio della Sopravvivenza nell’Infarto Miocardico (GISSI)-Prevenzione.. Circulation.

[r86] Martin JW, Whittle DM, Muir DC, Mabury SA (2004). Perfluoroalkyl contaminants in a food web from Lake Ontario.. Environ Sci Technol.

[r87] Mergler D, Anderson HA, Chan LH, Mahaffey KR, Murray M, Sakamoto M (2007). Methylmercury exposure and health effects in humans: a worldwide concern.. Ambio.

[r88] Michigan Department of Community Health (2007). Fish Consumption Survey of People Fishing and Harvesting Fish from the Saginaw Bay Watershed.. http://www.michigan.gov/documents/mdch/FCS_Final_rpt_061407_199288_7.pdf.

[r89] Morrison HA, Gobas F, Lazar R, Whittle DM, Haffner GD (1998). Projected changes to the trophodynamics of PCBs in the western Lake Erie ecosystem attributed to the presence of zebra mussels (*Dreissena polymorpha*).. Environ Sci Technol.

[r90] Morrissette J, Takser L, St-Amour G, Smargiassi A, Lafond J, Mergler D. (2004). Temporal variation of blood and hair mercury levels in pregnancy in relation to fish consumption history in a population living along the St. Lawrence River.. Environ Res.

[r91] Moses SK, Whiting AV, Muir DC, Wang X, O’Hara TM (2009). Organic nutrients and contaminants in subsistence species of Alaska: concentrations and relationship to food preparation method.. Int J Circumpolar Health.

[r92] Mozaffarian D, Rimm EB (2006). Fish intake, contaminants, and human health: evaluating the risks and the benefits.. JAMA.

[r93] National Fisheries Institute Inc (2008). Top 10 Consumed Seafoods.. http://www.aboutseafood.com/about/about-seafood/Top-10-Consumed-Seafoods.

[r94] O’Grady Milbrath M, Wenger Y, Chang CW, Emond C, Garabrant D, Gillespie BW (2009). Apparent half-lives of dioxins, furans, and polychlorinated biphenyls as a function of age, body fat, smoking status, and breast-feeding.. Environ Health Perspect.

[r95] Oken E, Kleinman KP, Berland WE, Simon SR, Rich-Edwards JW, Gillman MW (2003). Decline in fish consumption among pregnant women after a national mercury advisory.. Obstet Gynecol.

[r96] Oken E, Radesky JS, Wright RO, Bellinger DC, Amarasiriwardena CJ, Kleinman KP (2008). Maternal fish intake during pregnancy, blood mercury levels, and child cognition at age 3 years in a US cohort.. Am J Epidemiol.

[r97] Oken E, Wright RO, Kleinman KP, Bellinger D, Amarasiriwardena CJ, Hu H (2005). Maternal fish consumption, hair mercury, and infant cognition in a U.S. cohort.. Environ Health Perspect.

[r98] OMOE (Ontario Ministry of the Environment) (2011). Guide to Eating Ontario Sport Fish 2011–2012.. http://www.ene.gov.on.ca/environment/en/resources/STD01_078455.html.

[r99] Passos CJ, Mergler D (2008).

[r100] Persky V, Turyk M, Anderson HA, Hanrahan LP, Falk C, Steenport DN (2001). The effects of PCB exposure and fish consumption on endogenous hormones.. Environ Health Perspect.

[r101] Philibert A, Vanier C, Abdelouahab N, Chan HM, Mergler D (2006). Fish intake and serum fatty acid profiles from freshwater fish.. Am J Clin Nutr.

[r102] Quinn C, Wania F, Czub G, Breivik K. (2011). Investigating intergenerational differences in human PCB exposure due to variable emissions and reproductive behaviors.. Environ Health Perspect.

[r103] Rice DC (2008). Overview of modifiers of methylmercury neurotoxicity: chemicals, nutrients, and the social environment.. Neurotoxicology.

[r104] Schantz SL, Gasior DM, Polverejan E, McCaffrey RJ, Sweeney AM, Humphrey HE (2001). Impairments of memory and learning in older adults exposed to polychlorinated biphenyls via consumption of Great Lakes fish.. Environ Health Perspect.

[r105] Schell LM, Gallo MV (2010). Relationships of putative endocrine disruptors to human sexual maturation and thyroid activity in youth.. Physiol Behav.

[r106] Shimshack JP, Ward MB, Beatty TKM (2007). Mercury advisories: information, education, and fish consumption.. J Environ Econ Manage.

[r107] Sontrop JM, Campbell MK, Evers SE, Speechley KN, Avison WR (2007). Fish consumption among pregnant women in London, Ontario: associations with socio-demographic and health and lifestyle factors.. Can J Public Health.

[r108] Southwick Associates (2008). Today’s Angler.

[r109] State of the Great Lakes (2009). Contaminants in Whole Fish. Indicator.

[r110] Steenland K, Bertazzi P, Baccarelli A, Kogevinas M. (2004). Dioxin revisited: developments since the 1997 IARC classification of dioxin as a human carcinogen.. Environ Health Perspect.

[r111] Stewart PW, Lonky E, Reihman J, Pagano J, Gump BB, Darvill T (2008). The relationship between prenatal PCB exposure and intelligence (IQ) in 9-year-old children.. Environ Health Perspect.

[r112] Surette ME (2008). The science behind dietary omega-3 fatty acids.. CMAJ.

[r113] Swackhamer DL, Schottler S, Pearson RF (1999). Air-water exchange and mass balance of toxaphene in the Great Lakes.. Environ Sci Technol.

[r114] Tee PG, Sweeney AM, Symanski E, Gardiner JC, Gasior DM, Schantz SL (2003). A longitudinal examination of factors related to changes in serum polychlorinated biphenyl levels.. Environ Health Perspect.

[r115] Tilden J, Hanrahan LP, Anderson H, Palit C, Olson J, Kenzie WM (1997). Health advisories for consumers of Great Lakes sport fish: is the message being received?. Environ Health Perspect.

[r116] Tomasallo C, Anderson H, Haughwout M, Imm P, Knobeloch L. (2010). Mortality among frequent consumers of Great Lakes sport fish.. Environ Res.

[r117] Turyk M, Anderson H, Knobeloch L, Imm P, Persky V. (2009). Organochlorine exposure and incidence of diabetes in a cohort of Great Lakes sport fish consumers.. Environ Health Perspect.

[r118] USDA (U.S. Department of Agriculture) (2010). National Nutrient Database.. http://www.nal.usda.gov/fnic/foodcomp/search/.

[r119] U.S. EPA (U.S. Environmental Protection Agency) (2000). Guidance for Assessing Chemical Contaminant Data for Use in Fish Advisories. Vol 2. Risk Assessment and Fish Consumption Limits. 3rd ed.. http://www.epa.gov/waterscience/fish/advice/volume2/v2cover.pdf.

[r120] U.S. EPA (U.S. Environmental Protection Agency) and TERA (Toxicology Excellence for Risk Assessment) (1999). Comparative Dietary Risks: Balancing the Risks and Benefits of Fish Consumption. 5. Socio-Cultural Considerations of Fish Consumption.. http://www.tera.org/Publications/CDR%20Chapter5.pdf.

[r121] U.S. FDA (U.S. Food and Drug Administration) (2004). What You Need to Know about Mercury in Fish and Shellfish.. http://www.fda.gov/food/foodsafety/product-specificinformation/seafood/foodbornepathogenscontaminants/methylmercury/ucm115662.htm.

[r122] van der Voet H, de Mul A, van Klaveren JD (2007). A probabilistic model for simultaneous exposure to multiple compounds from food and its use for risk-benefit assessment.. Food Chem Toxicol.

[r123] Waller DP, Presperin C, Drum ML, Negrusz A, Larsen AK, van der Ven H (1996). Great Lakes fish as a source of maternal and fetal exposure to chlorinated hydrocarbons.. Toxicol Ind Health.

[r124] Wang YJ, Miller LA, Perren M, Addis PB (1990). Omega-3 fatty acids in Lake Superior fish.. J Food Sci.

[r125] Weisskopf MG, Anderson HA, Hanrahan LP, Kanarek MS, Falk CM, Steenport DM (2005). Maternal exposure to Great Lakes sport-caught fish and dichlorodiphenyl dichloroethylene, but not polychlorinated biphenyls, is associated with reduced birth weight.. Environ Res.

[r126] Welch AA, Bingham SA, Ive J, Friesen MD, Wareham NJ, Riboli E (2006). Dietary fish intake and plasma phospholipid n-3 polyunsaturated fatty acid concentrations in men and women in the European Prospective Investigation into Cancer-Norfolk United Kingdom cohort.. Am J Clin Nutr.

[r127] Wheatley B, Wheatley MA (2000). Methylmercury and the health of indigenous peoples: a risk management challenge for physical and social sciences and for public health policy.. Sci Total Environ.

[r128] Yokoyama M, Origasa H, Matsuzaki M, Matsuzawa Y, Saito Y, Ishikawa Y (2007). Effects of eicosapentaenoic acid on major coronary events in hypercholesterolaemic patients (JELIS): a randomised open-label, blinded endpoint analysis.. Lancet.

[r129] Zhu LY, Hites RA (2004). Temporal trends and spatial distributions of brominated flame retardants in archived fishes from the Great Lakes.. Environ Sci Technol.

