# Supplementary material for: Risks and Benefits of Consumption of Great Lakes Fish
Source: Environ Health Perspect. 2011 Sep 23;120(1):11–8. doi: 10.1289/ehp.1003396 (PMC3261933; doi:10.1289/ehp.1003396)
Supplement: (115 KB) PDF [file ehp.1003396.s001.pdf]

Supplemental Materials:

Risks and Benefits of Consumption of Great Lakes Fish

Mary E. Turyk[1], Satyendra P. Bhavsar[2], William Bowerman[3], Eric Boysen[4], Milton Clark[5], Miriam Diamond[6], Donna Mergler[7], Peter Pantazopoulos[8], Susan Schantz[9], and David O. Carpenter[10]

[1]Division of Epidemiology and Biostatistics, School of Public Health, University of Illinois at Chicago, Chicago, IL, USA

[2]Environmental Monitoring and Reporting Branch, Ontario Ministry of the Environment, Toronto, Ontario, Canada

[3]Department of Environmental Science and Technology, University of Maryland, College Park, MD, USA

[4]Great Lakes Branch, Ontario Ministry of Natural Resources, Peterborough, Ontario, Canada

[5]Division of Environmental and Occupational Health Sciences, School of Public Health, University of Illinois at Chicago, Chicago, IL, USA

[6]Department of Geography and Program in Planning and Department of Chemical Engineering and Applied Chemistry University of Toronto, Ontario, Canada

[7]Centre de Recherche Interdisciplinaire sur la Biologie, la Santé, la Société et l'Environnement (CINBIOSE), Université du Québec à Montreal, Canada

[8]Food Laboratories Division, Ontario Region, Health Canada, Toronto, Ontario, Canada

[9]Department of Veterinary Biosciences, University of Illinois at Urbana-Champaign, Urbana, IL, USA

[10]Institute for Health and the Environment, University at Albany, Rensselaer, NY, USA

Supplemental Material, Table 1: Comparison of Omega-3 Fatty Acids in Great Lakes and Marine Fish

Location	Sample	Species	N	Total Fat g/100 g fish	Omega-3 g/100 g lipid	Omega-3 mg/100 g fish	EPA g/100 g lipid	EPA mg/100 g fish	DHA g/100 g lipid	DHA mg/100 g fish	DHA+EPA g/100 g lipid	DHA+EPA mg/100 g fish	Author
Lake Superior	Fillet with skin	Chub	9	13.2	26.4	3485	5.4	713	5.9	779	11	1492	Wang et al. 1990[a]
		Herring	12	10.8	33.7	3640	4.9	529	5.3	572	10	1102	
		Sucker	3	2.8	28.2	790	7.7	216	7.2	202	15	417	
		Smelt	43	3.3	37.6	1241	8.6	284	7.5	248	16	531	
		Whitefish	4	7.2	32.5	2340	6.6	475	7.6	547	14	1022	
		Burbot	3	1	26	260	6.6	66	11.4	114	18	180	
		Lean Lake Trout	4	9.7	29.7	2881	4.6	446	8.2	795	13	1242	
		Siscowet Lake Trout	8	25.7	24.1	6194	4.8	1234	7	1799	12	3033	
		Mean		9.2	29.8	2604	6.2	495	7.5	632	13.7	1127	
		Median		8.5	29.0	2611	6.0	461	7.4	560	13.5	1062	
		Min		1.0	24.1	260	4.6	66	5.3	114	10.2	180	
		Max		25.7	37.6	6194	8.6	1234	11.4	1799	18.0	3033	
St Lawrence River	Fillet skinned defatted	Bullhead	9	1.5	17.8	267	NA	NA	NA	NA			Chan et al. 1999[a]
		Perch	8	1	27	270	NA	NA	NA	NA			
		Pike	10	1.3	29.1	378	NA	NA	NA	NA			
		Smallmouth Bass	10	2	14.6	292	NA	NA	NA	NA			
		Sturgeon	10	3.3	24.8	818	NA	NA	NA	NA			
		Walleye	10	1.1	5.7	63	NA	NA	NA	NA			
		Mean		1.7	19.8	348							
		Median		1.4	21.3	281							
		Min		1	5.7	63							
		Max		3.3	29.1	818							
Lake Superior	Oil from fillet skinned	Siscowet Trout	3	36	18.2	6552	3.9	1404	7.1	2556	11	3960	Karahadian and Lindsay 1989[a]
		Lake Trout	3	7	23.6	1652	4	280	9.6	672	14	952	
Lake Michigan		Lake Trout	3	11	20.2	2222	4.5	495	5.9	649	10	1144	
		Brook Trout	3	4	20.8	832	4.1	164	9.3	372	13	536	
		Mean		14.5	20.7	2815	4.1	586	8.0	1062	12.1	1648	
		Median		9.0	20.5	1937	4.1	388	8.2	661	12.2	1048	
		Min		4	18.2	832	3.9	164	5.9	372	10.4	536	
		Max		36	23.6	6552	4.5	1404	9.6	2556	13.6	3960	

Location	Sample	Species	N	Total Fat g/100 g fish	Omega-3 g/100 g lipid	Omega-3 mg/100 g fish	EPA g/100 g lipid	EPA mg/100 g fish	DHA g/100 g lipid	DHA mg/100 g fish	DHA+EPA g/100 g lipid	DHA+EPA mg/100 g fish	Author
Southeastern	Fillet	Black Sea Bass	2	0.7	NA	NA	4.9	27	23.4	121	28	148	Gooch et al. 1987
U.S. [c]	skinned	Bluefish	4	2.4	NA	NA	4.7	83	21.1	174	26	257	
		Channel Catfish	3	2.4	NA	NA	3.7	76	4.8	94	9	170	
		Atlantic Croaker	3	2.2	NA	NA	4.5	125	6.4	125	11	250	
		Dolphin	3	0.8	NA	NA	3.5	22	31.1	196	35	218	
		Southern Flounder	2	0.6	NA	NA	3.6	18	21.7	95	25	113	
		Goosefish (Monkfish)	4	0.6	NA	NA	7	25	24.4	90	31	115	
		Gag Grouper	3	2.2	NA	NA	3.4	65	15.4	276	19	341	
		Yellowedge Grouper	3	1.0	NA	NA	3.3	36	20.2	209	24	245	
		White Grunt	3	0.7	NA	NA	5.9	33	18	88	24	121	
		Speckled Hind	4	4.4	NA	NA	2.7	87	12.4	403	15	490	
		Crevalle Jack	4	3.9	NA	NA	3.8	113	16.2	258	20	371	
		Southern Kingfish	3	3.5	NA	NA	2.8	67	5.4	175	8	242	
		Ladyfish	3	4.4	NA	NA	2.5	83	7.5	251	10	334	
		King Mackerel	2	1.7	NA	NA	5.2	45	10.9	131	16	176	
		Striped Mullet	5	5.1	NA	NA	7.5	355	7.1	136	15	491	
		Red Porgy	2	1.0	NA	NA	3.7	21	27.3	193	31	214	
		American Shad	4	14.6	NA	NA	3.7	506	6.5	887	10	1393	
		Atlantic Sharpnose Shark	3	0.8	NA	NA	2.2	13	18.5	112	21	125	
		Lemon Shark	2	0.6	NA	NA	2.1	6	15.5	49	18	55	
		Scalloped Hammerhead Shark	2	0.6	NA	NA	2	11	15.1	75	17	86	
		Tiger Shark	2	0.6	NA	NA	1.6	7	11.3	52	13	59	
		Sheepshead	3	1.6	NA	NA	3.9	51	6.4	82	10	133	
		Red Snapper	3	1.2	NA	NA	3.4	40	18.7	216	22	256	

Location	Sample	Species	N	Total Fat g/100 g fish	Omega-3 g/100 g lipid	Omega-3 mg/100 g fish	EPA g/100 g lipid	EPA mg/100 g fish	DHA g/100 g lipid	DHA mg/100 g fish	DHA+EPA g/100 g lipid	DHA+EPA mg/100 g fish	Author
		Vermilion Snapper	3	0.7	NA	NA	3.2	14	26.3	104	30	118	
		Tilefish	2	1.0	NA	NA	2.8	22	20.3	147	23	169	
		Blueline Tilefish	3	3.2	NA	NA	2.8	60	13.8	284	17	344	
		Gray Triggerfish	4	0.7	NA	NA	4.2	24	24.4	155	29	179	
		Weakfish	3	1.7	NA	NA	3.8	66	13.2	233	17	299	
		Mean		2.2			3.7	72	16.0	187	19.7	259	
		Median		1.2			3.6	40	15.5	147	18.8	214	
		Min		0.6			1.6	6	4.8	49	8.2	55	
		Max		14.6			7.5	506	31.1	887	34.6	1393	
Commercial Fish Most Frequently	Edible portion	Salmon, Atlantic farmed		13.4	NA	NA	6.4	862	8.2	1104	15	1966	USDA 2010[b]
Consumed in US[d]		Salmon, Atlantic wild		6.3	NA	NA	5.1	321	17.6	1115	23	1436	
		Tuna, bluefin		4.9	NA	NA	5.8	283	18.2	890	24	1173	
		Tuna, skipjack		1.0	NA	NA	7.0	71	18.3	185	25	256	
		Cod, Atlantic		0.7	NA	NA	9.6	64	17.9	120	27	184	
		Pollock, Altantic		1.0	NA	NA	7.2	71	35.7	350	43	421	
		Tilapia		1.7	NA	NA	0.3	5	5.1	86	5	91	
		Catfish farmed		5.9	NA	NA	0.3	17	1.0	57	1	74	
		Catfish wild		2.8	NA	NA	4.6	130	8.3	234	13	364	
		Tuna, canned		0.8	NA	NA	5.7	47	27.2	223	33	270	
		Crab, blue		1.1	NA	NA	15.7	170	13.9	150	30	320	
		Shrimp		1.0	NA	NA	3.0	30	0.3	3	3	33	
		Lobster		0.8	NA	NA	13.6	102	9.1	68	23	170	
		Clams		1.0	NA	NA	4.5	43	6.7	64	11	107	
		Mean		2.2			6.3	104	13.8	273	20.1	377	
		Mean		1.0			5.8	71	11.5	168	22.7	263	
		Min		0.7			0.3	5	0.3	3	1.2	33	
		Max		6.3			15.7	321	35.7	1115	43.0	1436	

Location	Sample	Species	N	Total Fat g/100 g fish	Omega-3 g/100 g lipid	Omega-3 mg/100 g fish	EPA g/100 g lipid	EPA mg/100 g fish	DHA g/100 g lipid	DHA mg/100 g fish	DHA+EPA g/100 g lipid	DHA+EPA mg/100 g fish	Author
Commercial	Edible	Herring, Atlantic		9.0	NA	NA	7.9	709	9.6	862	17	1571	USDA 2010[b]
Fatty Marine	portion	Ancohvy		4.8	NA	NA	11.2	538	19.0	911	30	1449	
Fish		Mackerel, Atlantic		13.9	NA	NA	6.5	898	10.1	1401	17	2299	
		Salmon, Atlantic		6.3	NA	NA	5.1	321	17.6	1115	23	1436	
		Tuna, bluefin		4.9	NA	NA	5.8	283	18.2	890	24	1173	
		Mean		7.8			7.3	550	14.9	1036	22.2	1586	
		Median		6.3			6.5	538	17.6	911	22.6	1449	
		Min		4.8			5.1	283	9.6	862	16.5	1173	
		Max		13.9			11.2	898	19	1401	30.2	2299	

DHA=docosahexaenoic acid
EPA= eicosapentaenoic acid
NA=not available
[a] Calculated from mean values given in publication or database: omega-3 mg/100 g fish = omega-3 g/100 g total fat x total fat g/100 g fish x 1000 mg omega-3/1 g omega-3 / 100
[b] Calculated from mean values given in publication or database: omega-3 g/100 g total fat = omega-3 mg/100 g fish x 1 g omega-3/1000 mg omega-3 / total fat g/100 g fish x 100
[c] Charleston, SC seafood market, North Carolina coast
[d] Types of fish frequently consumed in the US as identified by the National Health and Nutrition Examination Survey (NHANES) (Mahaffey et al. 2008) and the National Marine Fisheries Service (National Fisheries Institute Inc 2008).
.

References

Chan HM, Trifonopoulos M, Ing A, Receveur O, Johnson E. 1999. Consumption of freshwater fish in Kahnawake: risks and benefits. Environ Res 80:S213-S222.

Gooch JA, Hale MB, Brown TJ, Bonnet JC, Brand CG, Regier LW. 1987. Proximate and fatty acid composition of 40 Southeastern U.S. finfish species. NOAA Technical Report NMFS 54:1-23.

Karahadian C, Lindsay RC. 1989. Composition of n-3 oils from some Great Lakes freshwater fish. J Food Composition Analysis 2:13-21.

Mahaffey KR, Clickner RP, Jeffries RA. 2008. Methylmercury and omega-3 fatty acids: co-occurrence of dietary sources with emphasis on fish and shellfish. Environ Res 107:20-29.

National Fisheries Institute Inc. 2008. Top 10 Consumed Seafoods. http://www.aboutseafood.com/about/about-seafood/Top-10-Consumed-Seafoods [accessed 12/18/08].

USDA. 2010. National Nutrient Database. http://www.nal.usda.gov/fnic/foodcomp/search/ [accessed 8 November 2008].

Wang YJ, Miller LA, Perren M, Addis PB. 1990. Omega-3 fatty acids in Lake Superior fish. J Food Sci 55:71-73.
